# Genome Sequencing of a Severe Acute Respiratory Syndrome Coronavirus 2 Isolate Obtained from a South African Patient with Coronavirus Disease 2019

**DOI:** 10.1128/MRA.00572-20

**Published:** 2020-07-02

**Authors:** Mushal Allam, Arshad Ismail, Zamantungwa T. H. Khumalo, Stanford Kwenda, Peter van Heusden, Ruben Cloete, Constantinos Kurt Wibmer, Phillip Senzo Mtshali, Florah Mnyameni, Thabo Mohale, Kathleen Subramoney, Sibongile Walaza, Wendy Ngubane, Nevashan Govender, Nkengafac V. Motaze, Jinal N. Bhiman

**Affiliations:** a National Institute for Communicable Diseases, National Health Laboratory Service, Johannesburg, South Africa; b South African Medical Research Council Bioinformatics Capacity Development Unit, South African National Bioinformatics Institute, University of the Western Cape, Cape Town, South Africa; c School of Public Health, Faculty of Health Sciences, University of the Witwatersrand, Johannesburg, South Africa; d Department of Global Health, Faculty of Medicine and Health Sciences, Stellenbosch University, Cape Town, South Africa; DOE Joint Genome Institute

## Abstract

As a contribution to the global efforts to track and trace the ongoing coronavirus pandemic, here we present the sequence, phylogenetic analysis, and modeling of nonsynonymous mutations for a severe acute respiratory syndrome coronavirus 2 (SARS-CoV-2) genome that was detected in a South African patient with coronavirus disease 2019 (COVID-19).

## ANNOUNCEMENT

Coronavirus disease 2019 (COVID-19), a disease caused by severe acute respiratory syndrome coronavirus 2 (SARS-CoV-2), a virus that belongs to the family *Coronaviridae* and the genus *Betacoronavirus* (1), is spreading rapidly in South Africa (https://sacoronavirus.co.za/), the rest of the African continent (https://africacdc.org/covid-19/), and the world (https://www.who.int/emergencies/diseases/novel-coronavirus-2019). We report here a complete genome sequence of a SARS-CoV-2 isolate obtained from a South African patient who had returned to South Africa after traveling to Italy.

Combined nasopharyngeal and oropharyngeal swabs were collected, and total nucleic acid extraction was performed using the MagNA Pure 96 DNA and viral nucleic acid (NA) small-volume kit (Roche, Switzerland) as described by the manufacturer to confirm the presence of SARS-CoV-2 using the TIB Molbiol LightMix Sarbeco E-gene real-time PCR assay (2). Ethical clearance was obtained from the Human Research Ethics Committee at University of the Witwatersand, Johannesburg, South Africa (protocol number M160667).

Metagenomic next-generation sequencing libraries were prepared from the viral RNA extracted from the samples. Total RNA quantity and integrity were assessed using a Qubit RNA assay kit (Invitrogen, USA) and a 4200 TapeStation instrument (Agilent Technologies, Germany). Host rRNA depletion was performed using a NEBNext rRNA depletion kit (New England Biolabs, USA) following the manufacturer’s instructions. Approximately 2 μg RNA was used for cDNA synthesis using a Maxima H minus double-stranded cDNA synthesis kit (Thermo Fisher Scientific, USA) primed with random hexamers. The paired-end libraries were prepared using the Nextera DNA Flex library preparation kit, followed by 2 × 300-bp sequencing on a MiSeq system (Illumina, USA).

**FIG 1:**
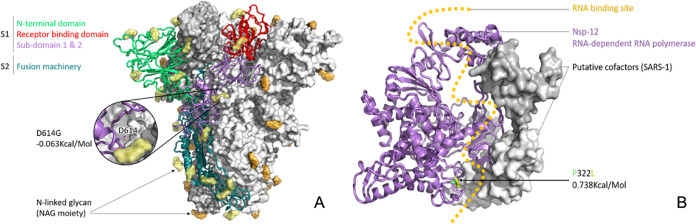
(A) SARS-CoV-2 spike (S) trimer modelled with SWISS-MODEL (9) using 6VXX structure as a template, drawn and colored in PyMol (https://pymol.org/2/). Domains of a single S1 protomer are shown in cartoon view and colored green (N-terminal domain, NTD), red (C-terminal domain/receptor binding domain, CTD/RBD), and purple (subdomains 1 and 2, SD1 and SD2). S2 is shown in dark teal, while *N*-acetylglucosamine moieties are colored yellow (cartoon protomer) or orange (surface protomers). The enlarged inset shows the location of D614, which is where a mutation has arisen in the R03006/20 South African strain, buried in the interprotomer interface. (B) SARS-CoV-2 nsp12 (RNA-dependent RNA polymerase, RDRP) modelled with SWISS-MODEL (9), drawn and colored in PyMol, based on the nsp12, nsp7, and nsp8 protein complex of SARS-1 (PDB ID 6NUR). The RNA binding groove is indicated (orange), with the adjacent P322 (green) to L322 (yellow) mutation shown in stick view.

The obtained metagenomic sequences (9,406,678 reads) were quality trimmed (Q > 20) using Trim Galore v0.6.5 (https://www.bioinformatics.babraham.ac.uk/projects/trim_galore/), and subsequently, FastQ Screen v0.14.0 (https://www.bioinformatics.babraham.ac.uk/projects/fastq_screen/) was used to filter out human and PhiX reads based on prebuilt Bowtie2 indexes for the human reference genome (GRCh38; ftp://ftp.ccb.jhu.edu/pub/data/bowtie_indexes/) and PhiX NCBI reference sequences (GenBank accession number NC_001422.1). To generate the consensus sequence, the remaining reads (23,489 reads) were then mapped to the complete genome of SARS-CoV-2 Wuhan-Hu-1 (GenBank accession number MN908947.3) using CLC Genomics Workbench v20. The complete genome size was 29,903 bp with a GC content of 38.00%. To identify the variants, the consensus sequence was combined with a collection of 965 SARS-CoV-2 genomes downloaded from the Global Initiative on Sharing All Influenza Data (GISAID) (3), and a multiple sequence alignment was generated using MAFFT v7.042 (4). From an initial list of 74 variants, 6 were confirmed by the evidence from mapped reads and retained. The average depth of coverage over the genome was 10 reads per fragment as determined by SAMtools v1.9 (5). Regions of high coverage (greater than 5 reads) were identified using covtobed v1.1.0 (6), and the resultant Browser Extensible Data (BED) file from covtobed was used to produce an interval tree (in Python), and the interval tree, in turn, was used to mask out variants in low-coverage regions (7). This masking confirmed that the 6 previously mentioned high-quality variants were located within the 76% of the genome that was covered by reads to a depth of greater than 5 reads and where the allele frequency for the variant allele was >60% (Table 1). The variants at 13,620 bp and 21,595 bp were not found in any other SARS-CoV-2 genome that was present in GISAID at the time that this report was drafted (1 April 2020). The impact of the spike protein D614G variant (Fig. 1A) and the P322L variant on the nsp12 protein (Fig. 1B) were predicted by the DUET Web server (8) to have slightly destabilizing and stabilizing effects, respectively. Neither the receptor binding domain of the spike protein nor the points of contact between the nsp12 and the putative SARS-CoV-2 cofactors nsp7 and nsp8 were impacted by these variants, leading to the assumption that, overall, the variants will not have a substantial effect on protein structure or function. All tools were run with default parameters unless otherwise indicated.

**TABLE 1:** Summary of the variants identified in the genome

Genomic position	Nucleotide change	No. of reads supporting/no. of reads mapped	Gene name	Amino acid change
241	C → T	15/16	5′ untranslated region	Synonymous
3037	C → T	13/13	ORF1ab/nsp3	Synonymous 193Phe
13620	C → T	6/6	ORF1ab/nsp12	Synonymous 58Asp
14408	C → T	18/18	ORF1ab/nsp12	Pro321Leu (P321L)
21595	C → T	7/7	Spike protein	Synonymous 10Val
23403	A → G	6/6	Spike protein	Asp614Gly (D614G)

### Data availability.

This sequence has been deposited in GenBank under the accession number MT324062 and at the GISAID EpiCoV under the identifier EPI_ISL_417186. The accession numbers for the Illumina MiSeq sequence raw reads in the NCBI Sequence Read Archive (SRA) are PRJNA624358 (BioProject), SRR11524818 and SRR11524819 (SRA), and SAMN14574670 and SAMN14574671 (BioSample).
